# Supplementary material for: Synthesis of New AIEE-Active Chalcones for Imaging of Mitochondria in Living Cells and Zebrafish In Vivo
Source: Int J Mol Sci. 2021 Aug 19;22(16):8949. doi: 10.3390/ijms22168949 (PMC8396511; doi:10.3390/ijms22168949)
Supplement: Supplementary file 1 [file ijms-22-08949-s001.zip › ijms-1325199-supplementary.pdf]

International Journal of *Molecular Sciences*

MDPI

Supplementary Information

Synthesis of New AIEE-Active Chalcones for Imaging of Mitochondria in Living Cells and Zebrafish In Vivo

Huiqing Luo [1], Na Li [1], Liyan Liu [1], Huaqiao Wang [2] and Feng He [1,*]

[1] School of Pharmaceutical Science, Sun Yat-sen University, Guangzhou 510006, China; luohq8@mail2.sysu.edu.cn (H.L.); lina49@mail2.sysu.edu.cn (N.L.); liuly37@mail2.sysu.edu.cn (L.L.)

[2] Department of Anatomy and Neurobiology, Zhongshan School of Medicine, Sun Yat-sen University, Guangzhou 510006, China; wanghq@mail.sysu.edu.cn

* Correspondence: hefeng@mail.sysu.edu.cn; Tel.: +86-203-994-3036

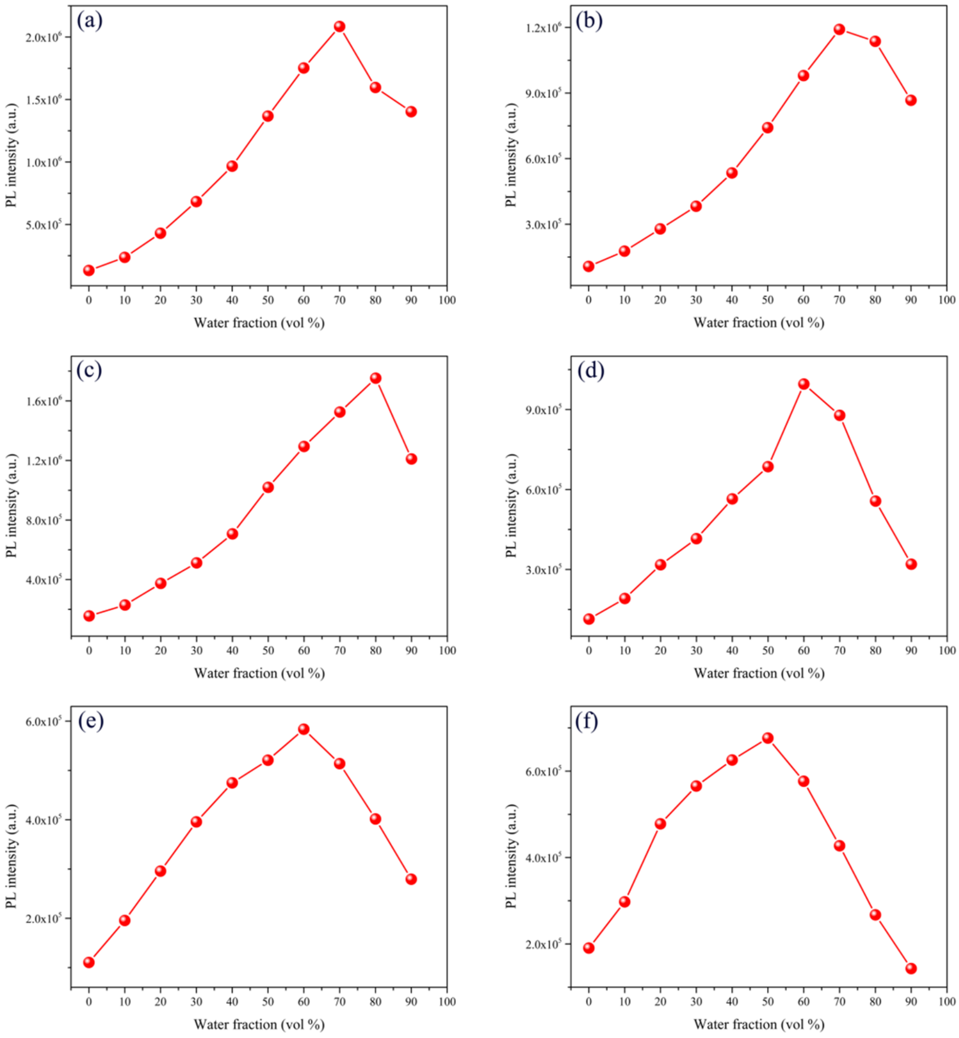

Figure S1. The variation trend of the maximum fluorescence intensity of compounds (**a**) **1**, (**b**) **2**, (**c**) **3**, (**d**) **4**, (**e**) **5**, (**f**) **6** in CH_3OH/H_2O mixed solutions ($c = 2.07 \times 10^{-5}$ M) with different water fractions (0%–90%).

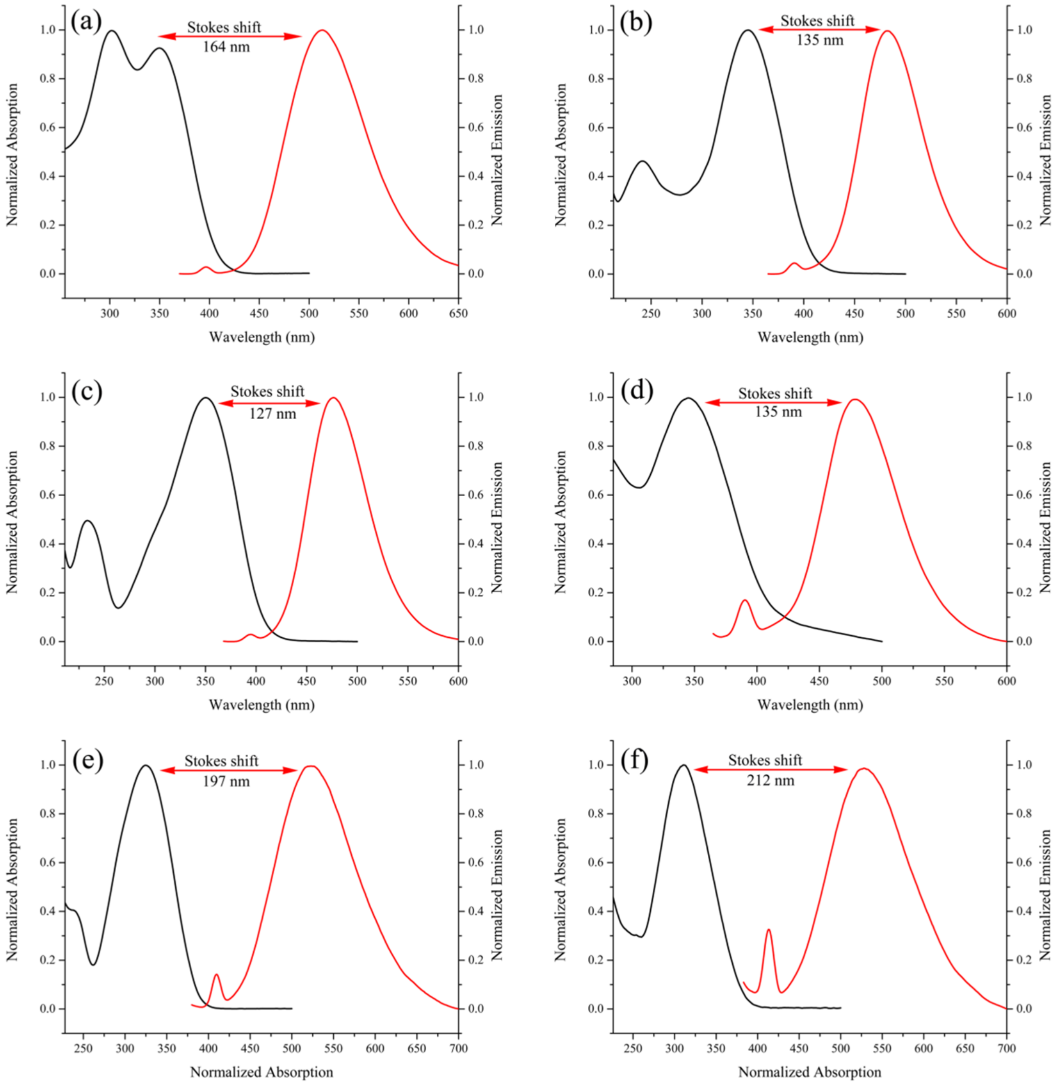

Figure S2. Normalized UV absorption and fluorescence spectra of compounds (**a**) **1**, (**b**) **2**, (**c**) **3**, (**d**) **4**, (**e**) **5**, (**f**) **6** in CH_3OH/H_2O mixed solutions ($c = 2.07 \times 10^{-5}$ M) with 90% water fractions.

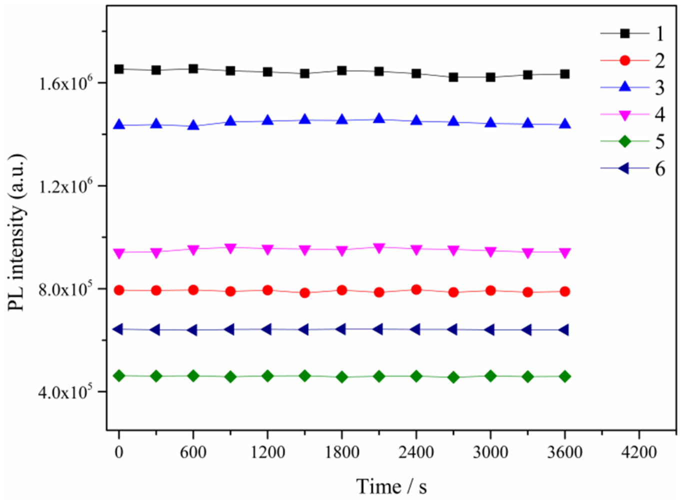

Figure S3. Time-dependent fluorescence spectra of **1** (3:7 *v:v*), **2** (3:7 *v:v*), **3** (2: 8 *v:v*) , **4** (4: 6 *v:v*), **5** (4: 6 *v:v*) and **6** (5:5 *v:v*) in CH_3OH/H_2O (*v/v*) mixed solutions ($c = 2.07 \times 10^{-5}$ M).

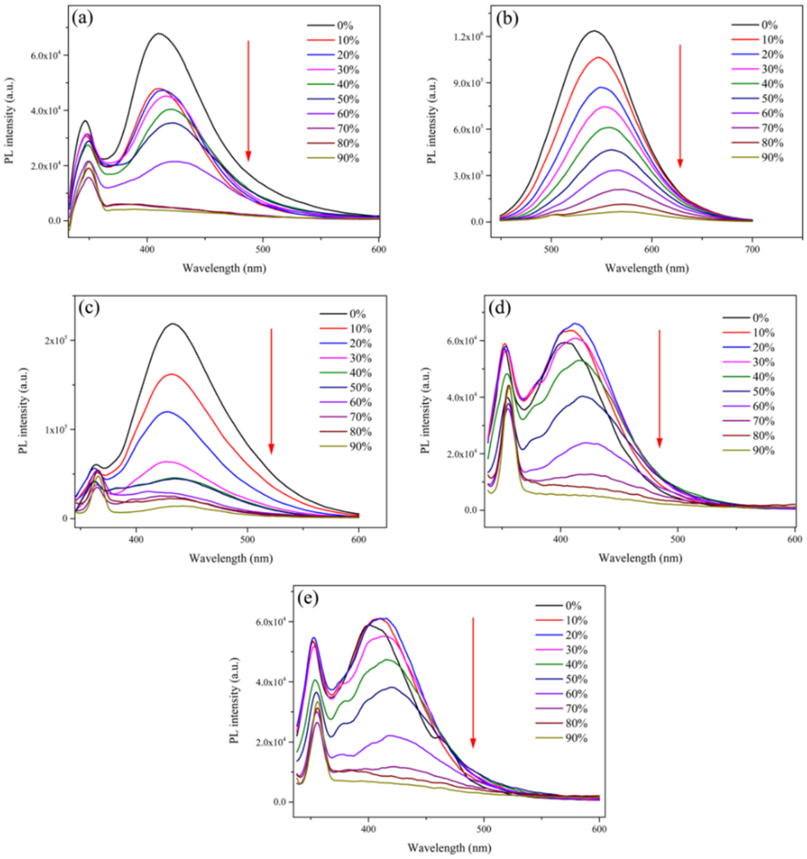

Figure S4. PL spectra of compounds (**a**) **7**, (**b**) **8**, (**c**) **9**, (**d**) **10**, (**e**) **11** in CH_3OH/H_2O mixed solutions ($c = 2.07 \times 10^{-5}$ M) with different water fractions (0%-90%).

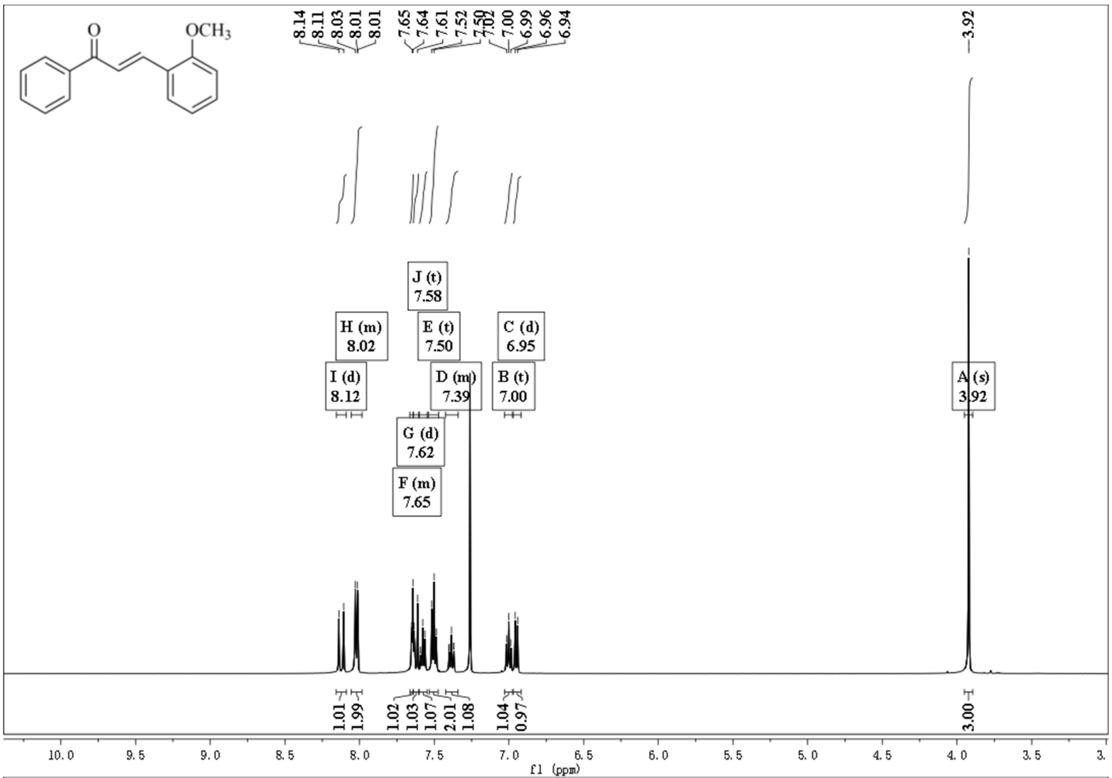
OCH3
O
8.14
8.11
8.03
8.01
8.01
7.65
7.64
7.61
7.52
7.50
7.02
7.00
6.99
6.96
6.94
3.92
J (t) 7.58
H (m) 8.02
E (t) 7.50
C (d) 6.95
I (d) 8.12
D (m) 7.39
B (t) 7.00
A (s) 3.92
G (d) 7.62
F (m) 7.65
1.01
1.99
1.02
1.03
1.07
2.01
1.08
1.04
0.97
3.00
f1 (ppm)

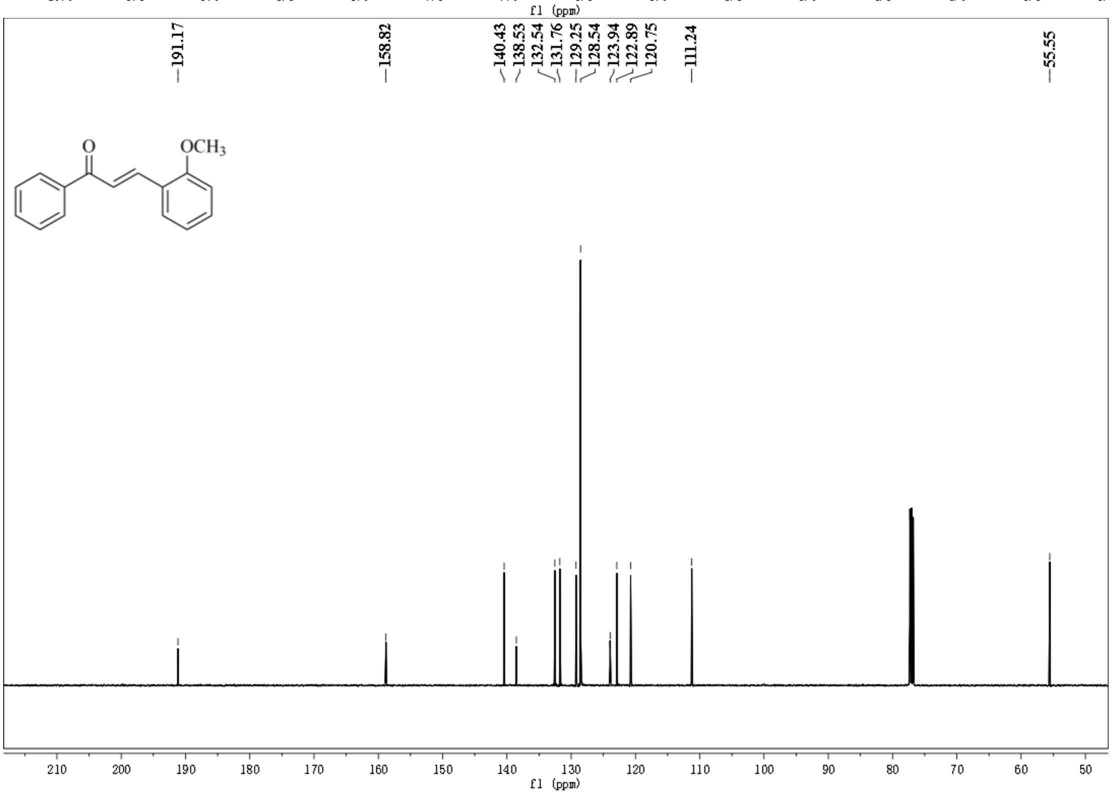
191.17
158.82
140.43
138.53
132.54
131.76
129.25
128.54
123.94
122.89
120.75
111.24
55.55
OCH3
O
f1 (ppm)

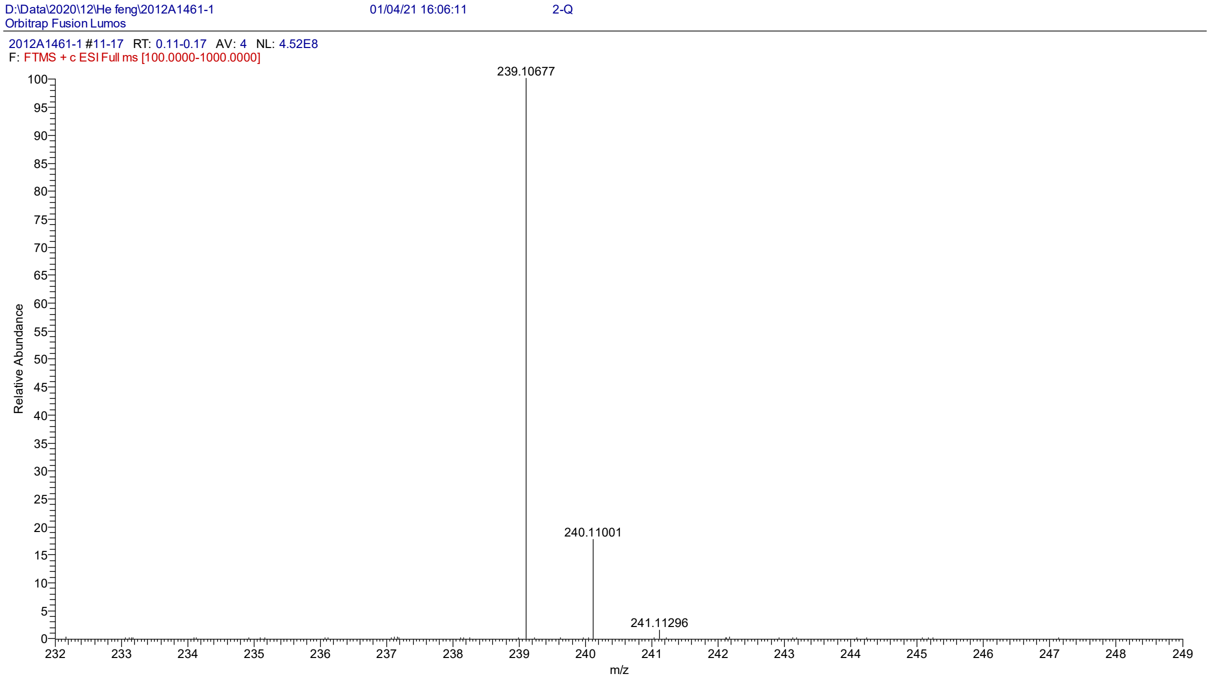

Figure S5. ^{1}H, ^{13}C NMR spectra and ESI-MS analysis for compound **1**.

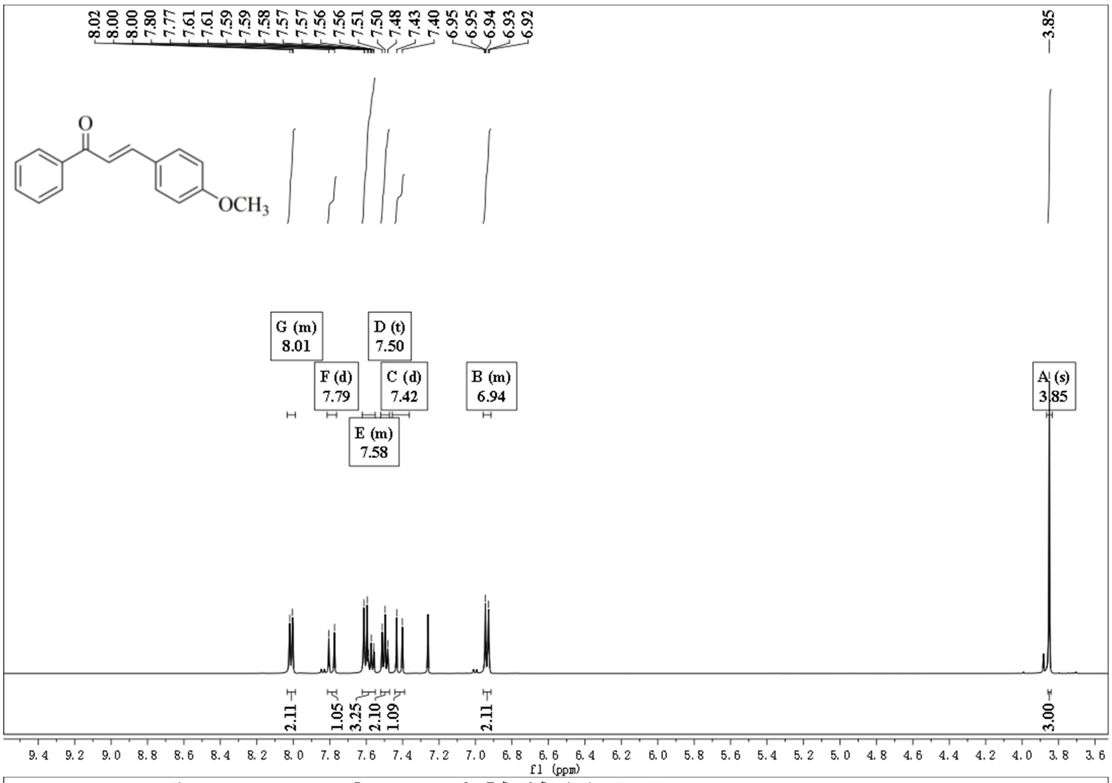
8.02 8.00 8.00 7.80 7.77 7.61 7.61 7.59 7.59 7.58 7.57 7.57 7.56 7.56 7.51 7.50 7.48 7.43 7.40 6.95 6.95 6.94 6.93 6.92
3.85
OCH3
G (m) 8.01
D (t) 7.50
F (d) 7.79
C (d) 7.42
B (m) 6.94
A (s) 3.85
E (m) 7.58
2.11 1.05 3.25 2.10 1.09 2.11 3.00
9.4 9.2 9.0 8.8 8.6 8.4 8.2 8.0 7.8 7.6 7.4 7.2 7.0 6.8 6.6 6.4 6.2 6.0 5.8 5.6 5.4 5.2 5.0 4.8 4.6 4.4 4.2 4.0 3.8 3.6
f1 (ppm)

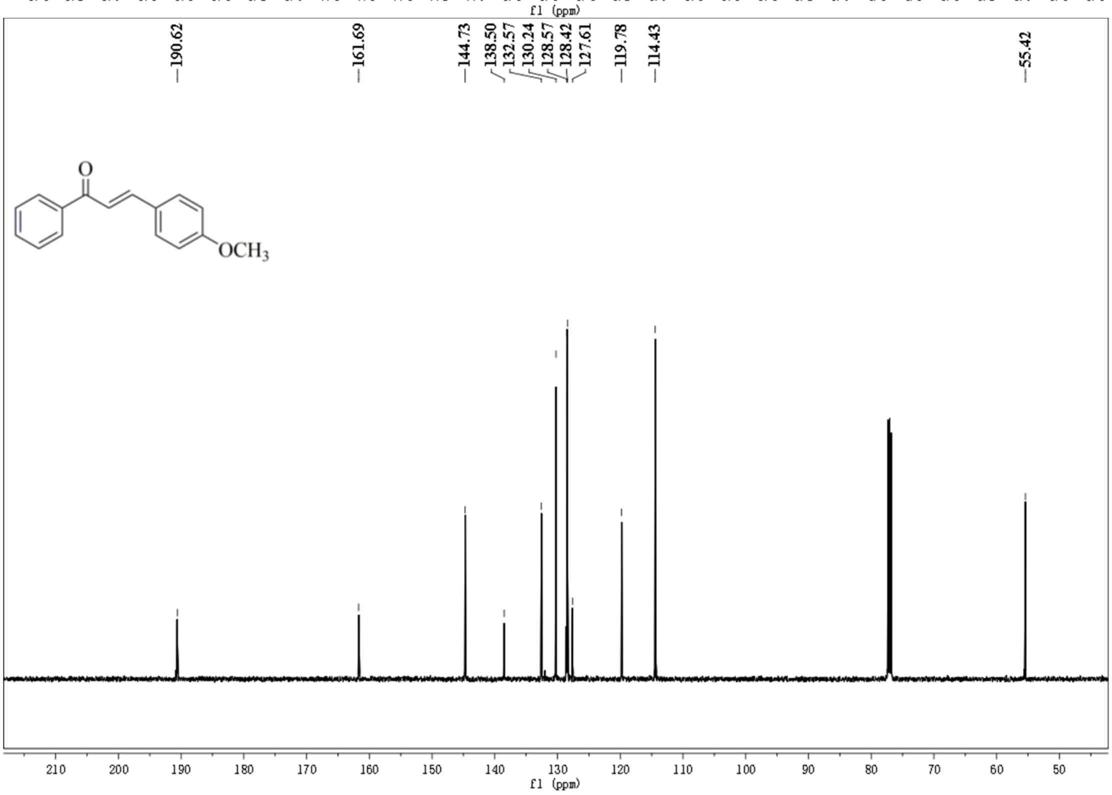
190.62
161.69
144.73 138.50 132.57 130.24 128.57 128.42 127.61
119.78 114.43
55.42
OCH3
210 200 190 180 170 160 150 140 130 120 110 100 90 80 70 60 50
f1 (ppm)

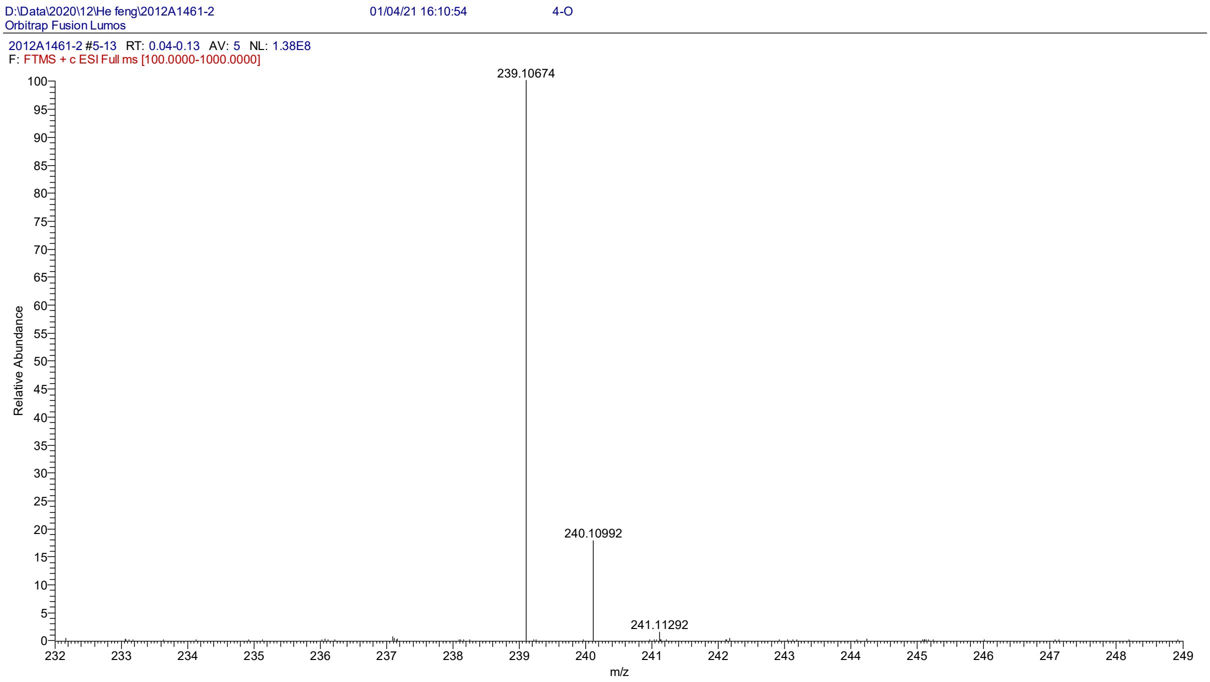

Figure S6. ^{1}H, ^{13}C NMR spectra and ESI-MS analysis for compound **2**.

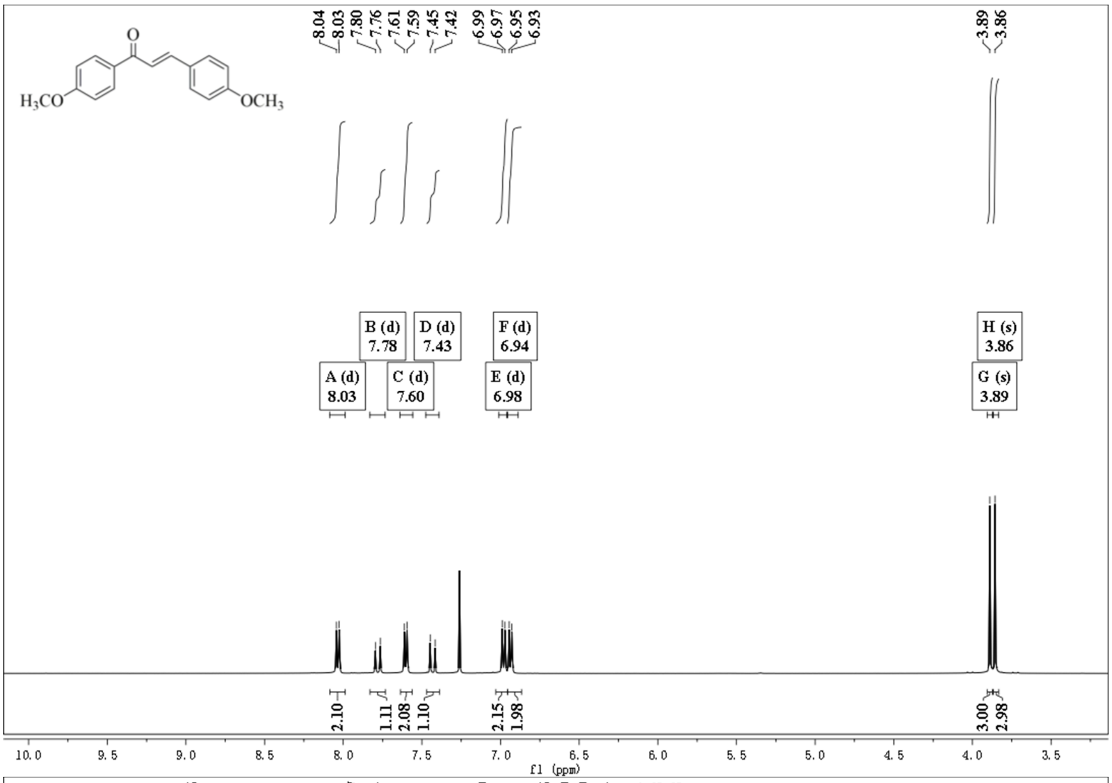
H3CO
OCH3
8.04
8.03
7.80
7.76
7.61
7.59
7.45
7.42
6.99
6.97
6.95
6.93
3.89
3.86
A (d) 8.03
B (d) 7.78
C (d) 7.60
D (d) 7.43
E (d) 6.98
F (d) 6.94
G (s) 3.89
H (s) 3.86
2.10
1.11
2.08
1.10
2.15
1.98
3.00
2.98
f1 (ppm)

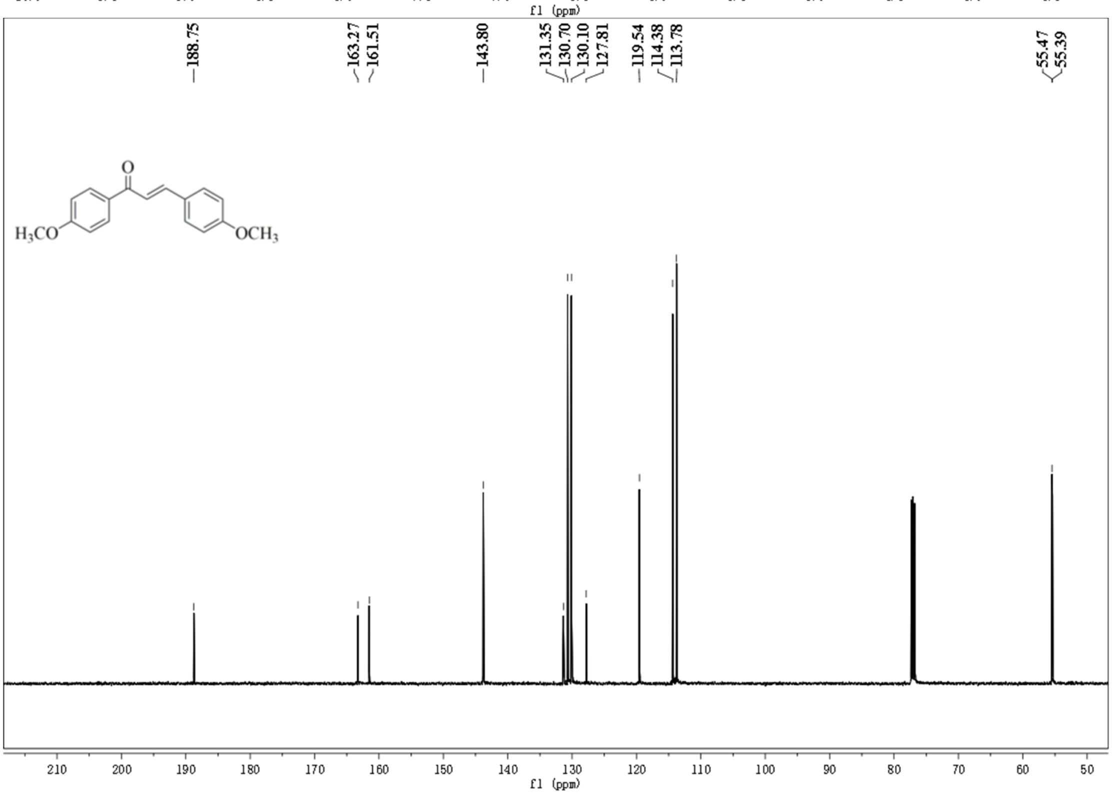
188.75
163.27
161.51
143.80
131.35
130.70
130.10
127.81
119.54
114.38
113.78
55.47
55.39
H3CO
OCH3
f1 (ppm)

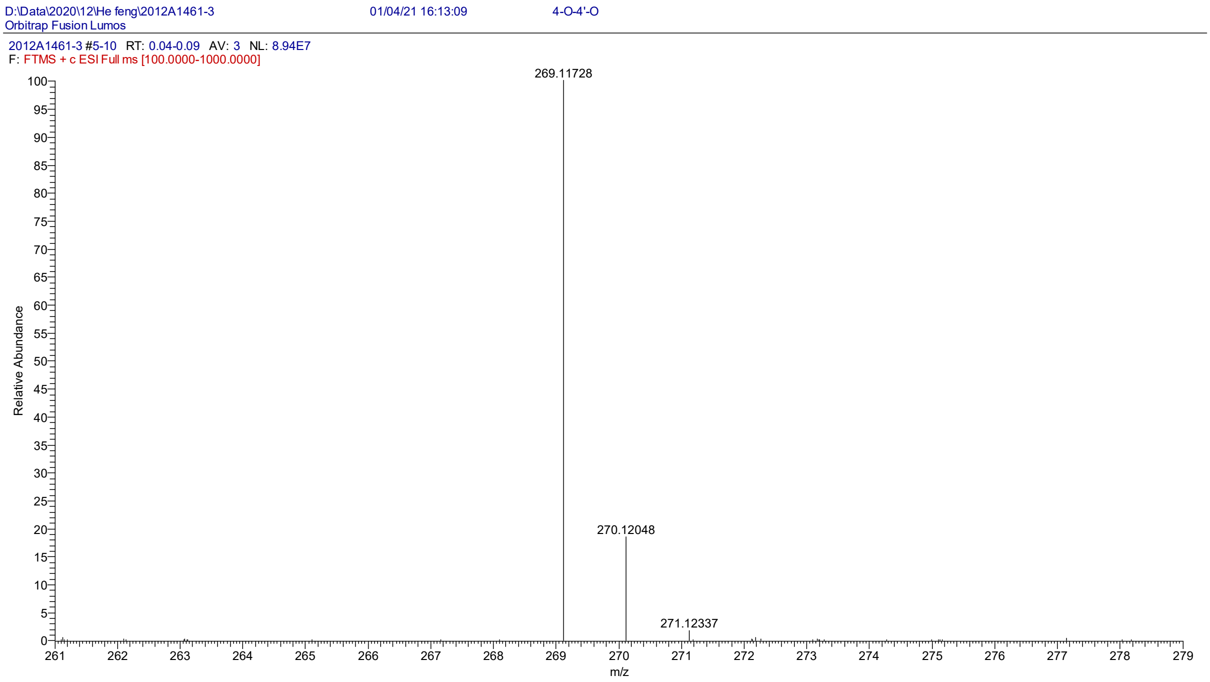

Figure S7. ^{1}H, ^{13}C NMR spectra and ESI-MS analysis for compound **3**.

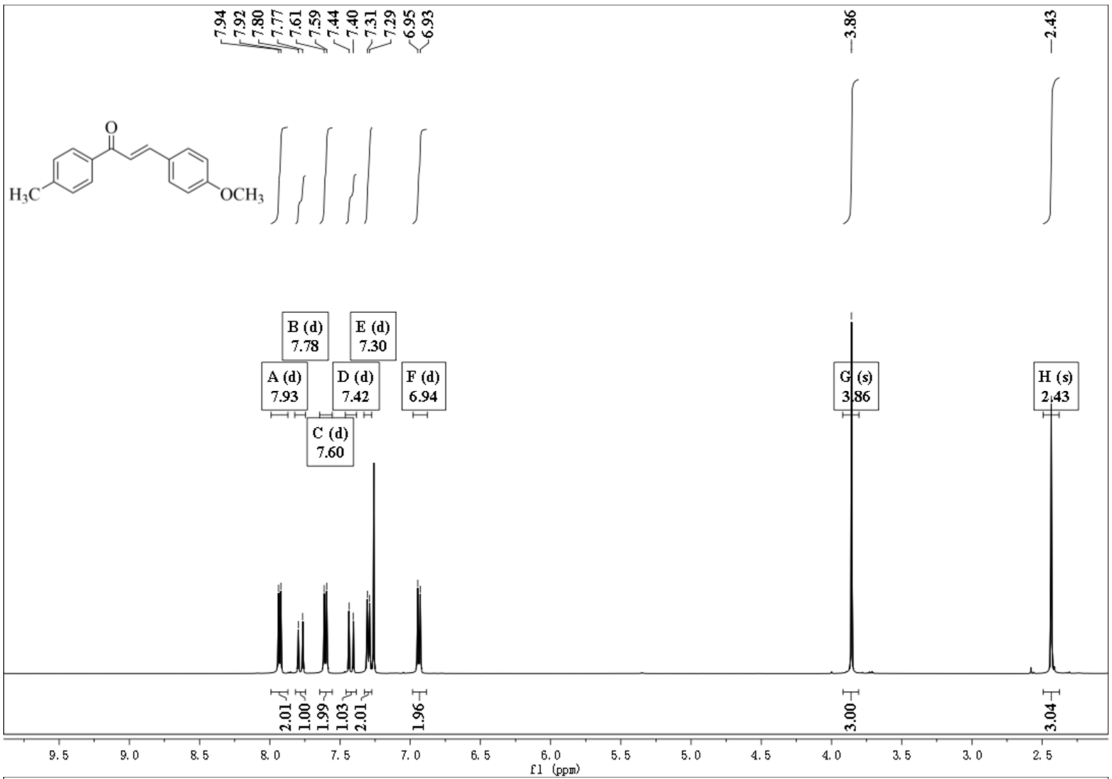
7.94
7.92
7.80
7.77
7.61
7.59
7.44
7.40
7.31
7.29
6.95
6.93
3.86
2.43
H3C
O
OCH3
B (d)
7.78
E (d)
7.30
A (d)
7.93
D (d)
7.42
F (d)
6.94
C (d)
7.60
G (s)
3.86
H (s)
2.43
2.01
1.00
1.99
1.03
2.01
1.96
3.00
3.04
9.5
9.0
8.5
8.0
7.5
7.0
6.5
6.0
5.5
5.0
4.5
4.0
3.5
3.0
2.5
f1 (ppm)

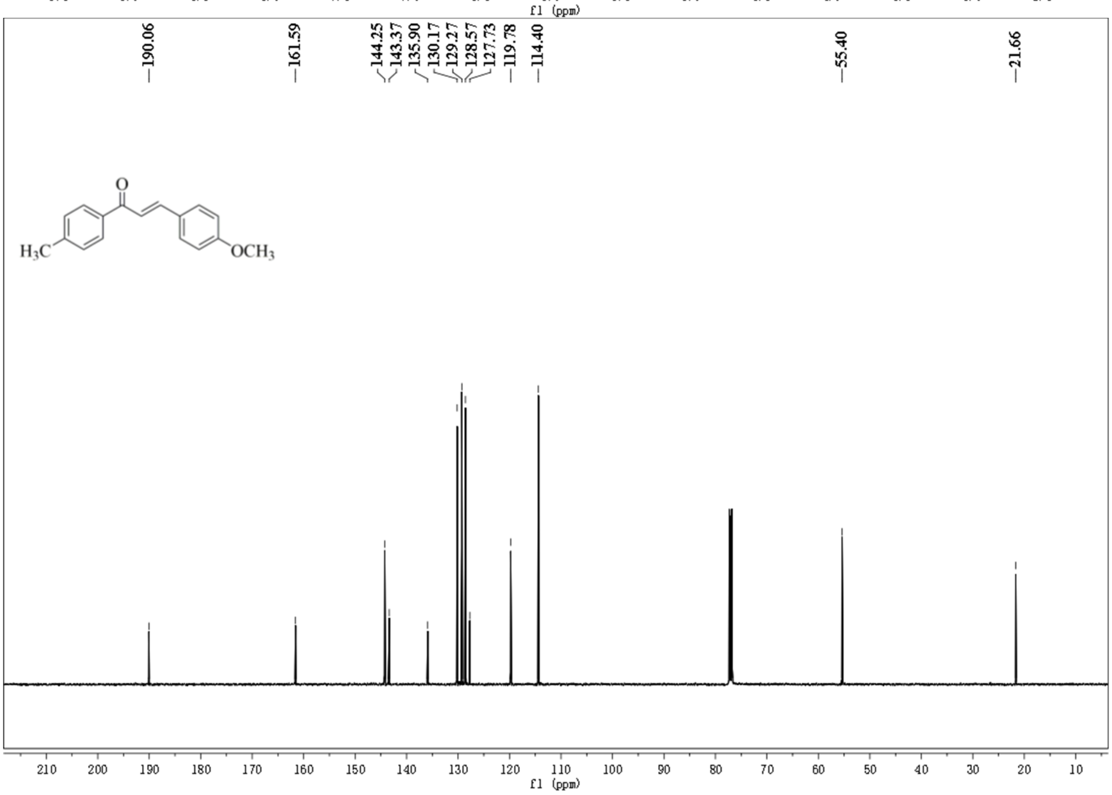
190.06
161.59
144.25
143.37
135.90
130.17
129.27
128.57
127.73
119.78
114.40
55.40
21.66
H3C
O
OCH3
210
200
190
180
170
160
150
140
130
120
110
100
90
80
70
60
50
40
30
20
10
f1 (ppm)

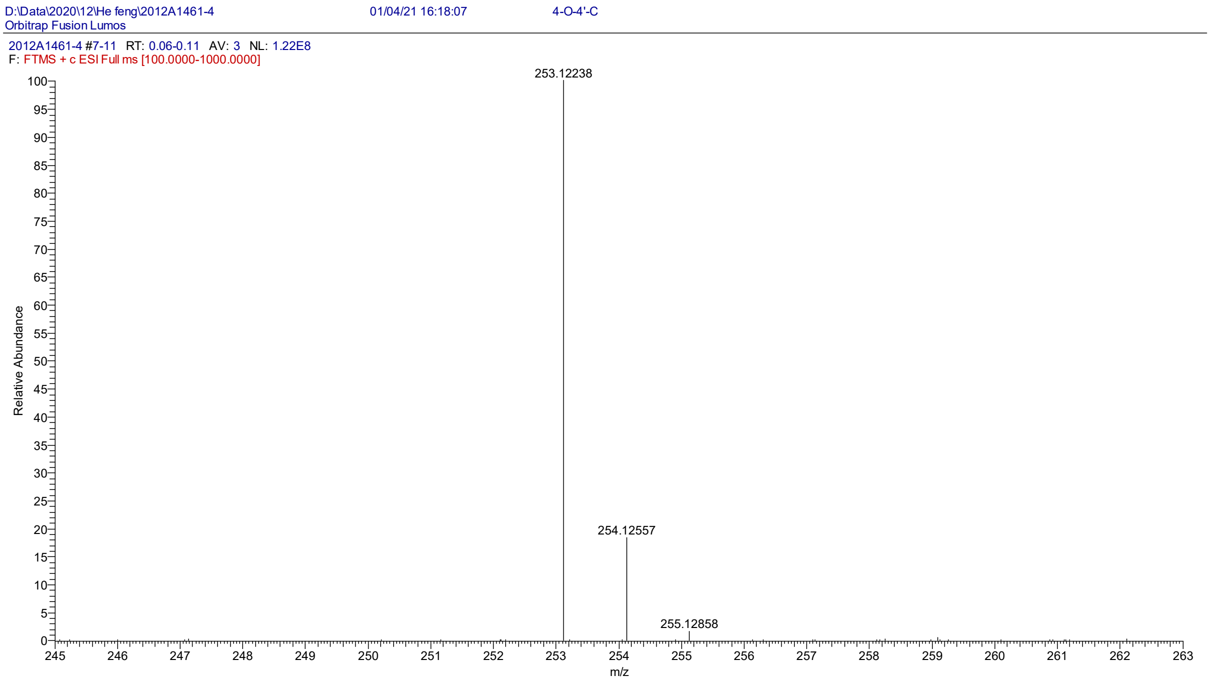

Figure S8. ^{1}H, ^{13}C NMR spectra and ESI-MS analysis for compound **4**.

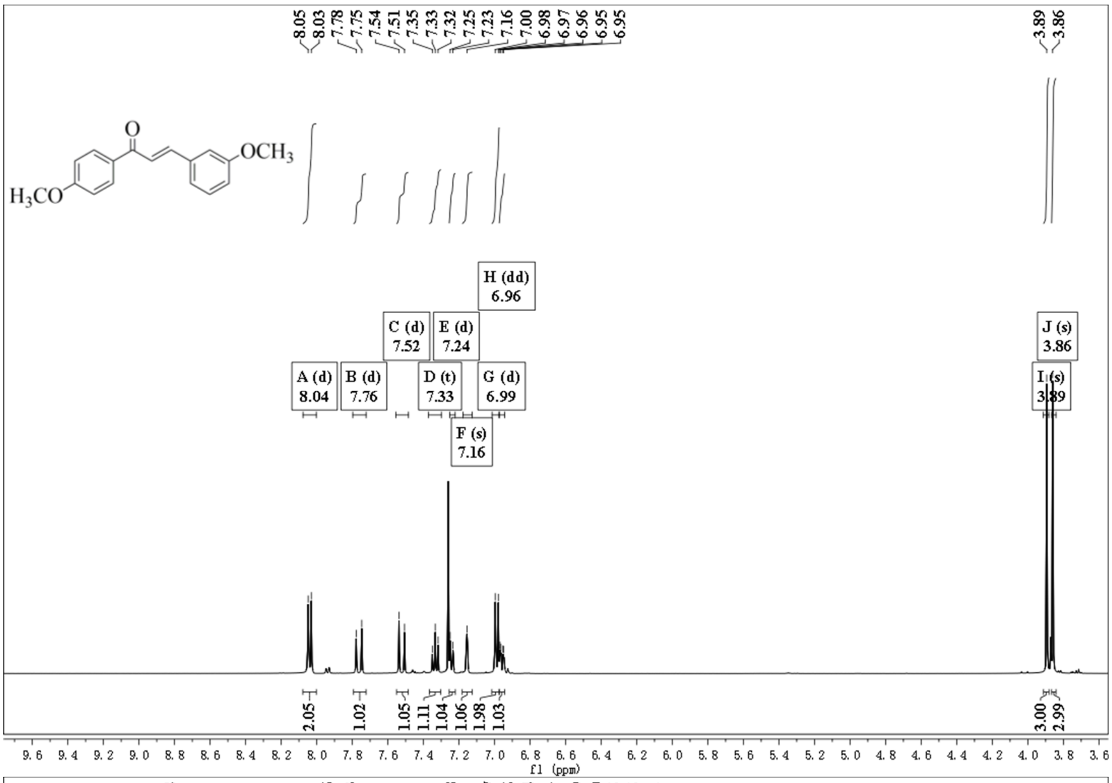
8.05
8.03
7.78
7.75
7.54
7.51
7.35
7.33
7.32
7.25
7.23
7.16
7.00
6.98
6.97
6.96
6.95
6.95
3.89
3.86
O
OCH3
H3CO
H (dd)
6.96
C (d)
7.52
E (d)
7.24
J (s)
3.86
A (d)
8.04
B (d)
7.76
D (t)
7.33
G (d)
6.99
I (s)
3.89
F (s)
7.16
2.05
1.02
1.05
1.11
1.04
1.06
1.98
1.03
3.00
2.99
f1 (ppm)

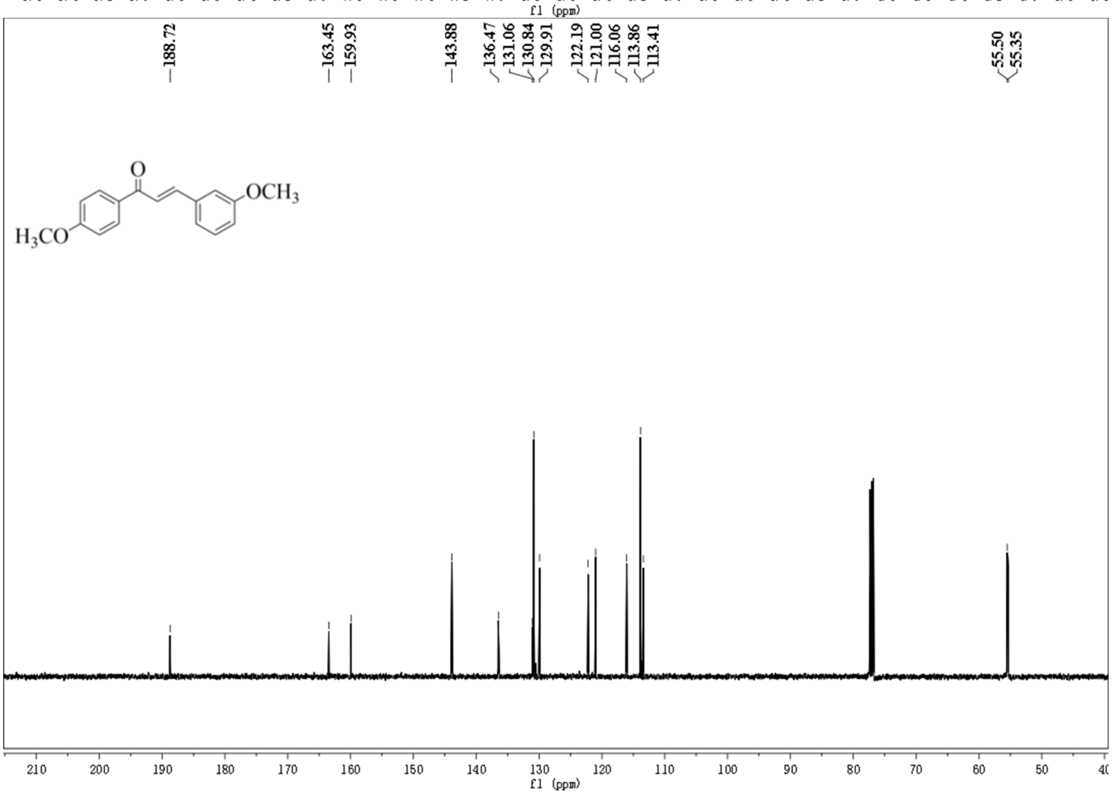
188.72
163.45
159.93
143.88
136.47
131.06
130.84
129.91
122.19
121.00
116.06
113.86
113.41
55.50
55.35
O
OCH3
H3CO
f1 (ppm)

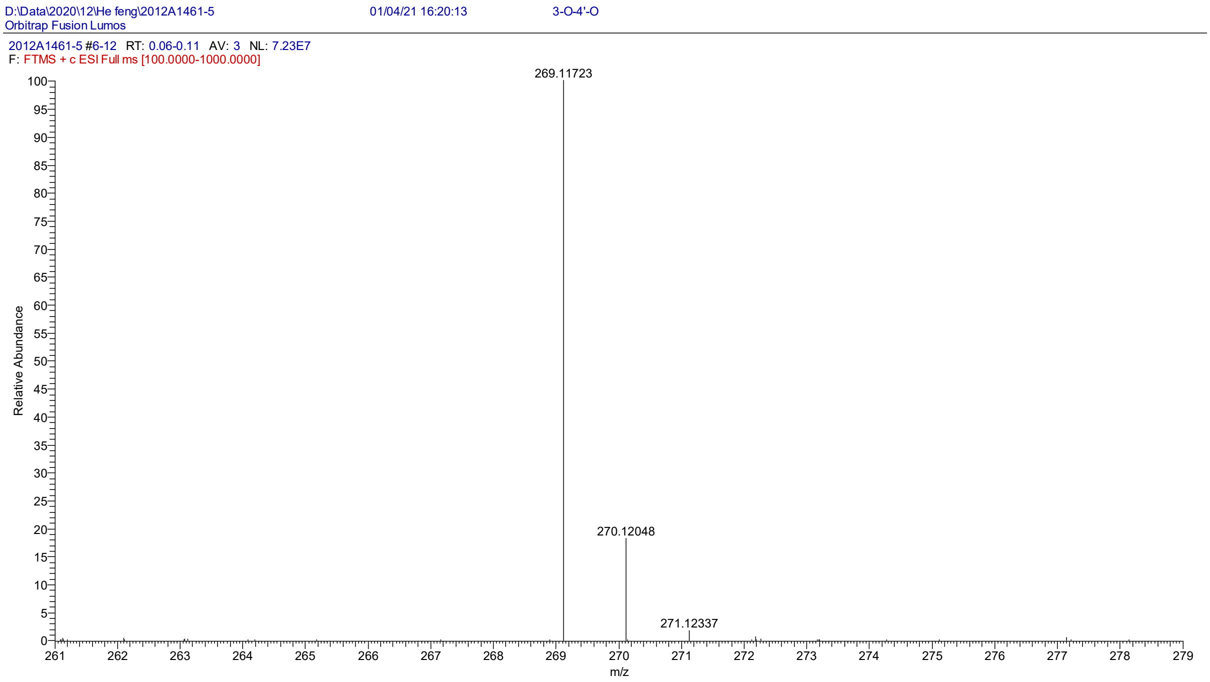

Figure S9. ^{1}H, ^{13}C NMR spectra and ESI-MS analysis for compound **5**.

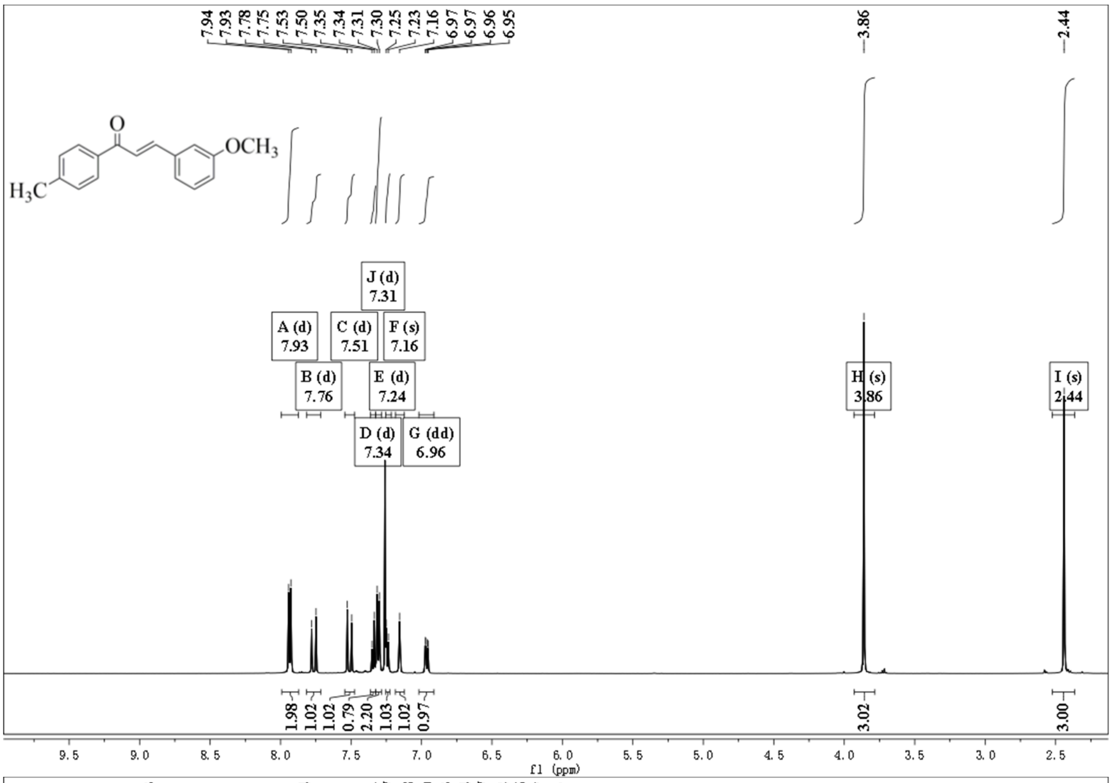
7.94 7.93 7.78 7.75 7.53 7.50 7.35 7.34 7.31 7.30 7.25 7.23 7.16 6.97 6.97 6.96 6.95
3.86
2.44
O
OCH3
H3C
J (d) 7.31
A (d) 7.93
C (d) 7.51
F (s) 7.16
B (d) 7.76
E (d) 7.24
H (s) 3.86
I (s) 2.44
D (d) 7.34
G (dd) 6.96
1.98 1.02 1.02 0.79 2.20 1.03 1.02 0.97
3.02
3.00
9.5 9.0 8.5 8.0 7.5 7.0 6.5 6.0 5.5 5.0 4.5 4.0 3.5 3.0 2.5
f1 (ppm)

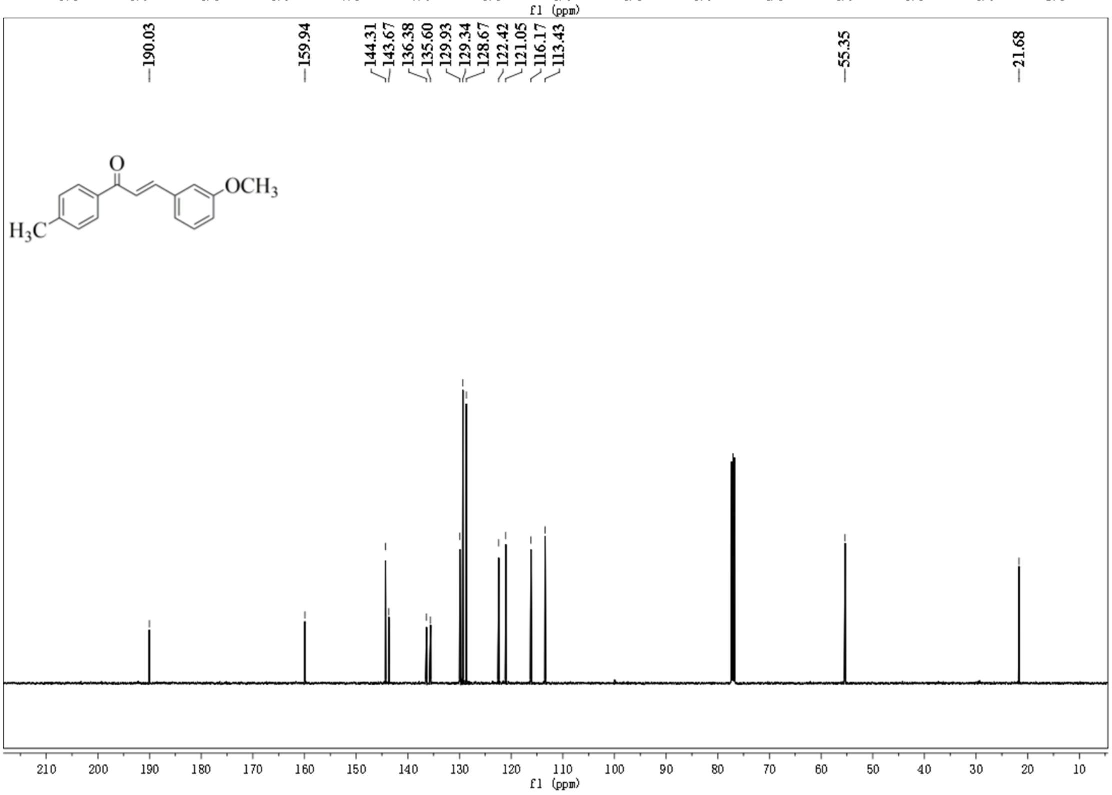
190.03
159.94
144.31 143.67 136.38 135.60 129.93 129.34 128.67 122.42 121.05 116.17 113.43
55.35
21.68
O
OCH3
H3C
210 200 190 180 170 160 150 140 130 120 110 100 90 80 70 60 50 40 30 20 10
f1 (ppm)

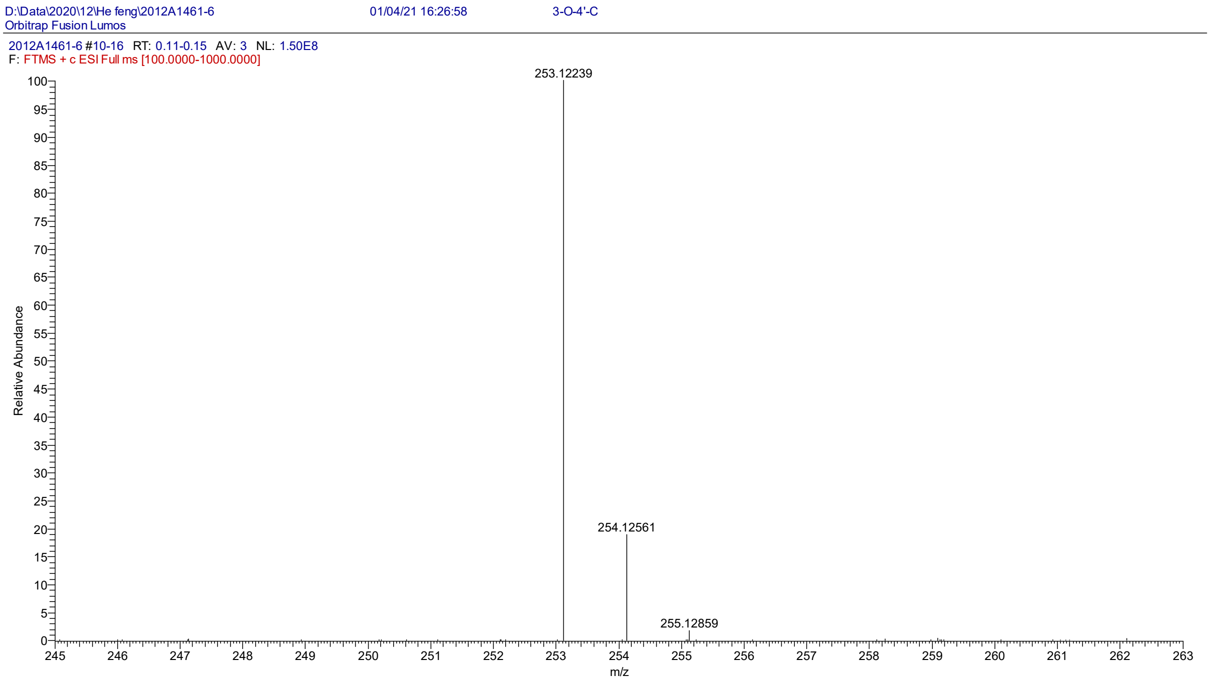

Figure S10. ^{1}H, ^{13}C NMR spectra and ESI-MS analysis for compound **6**.

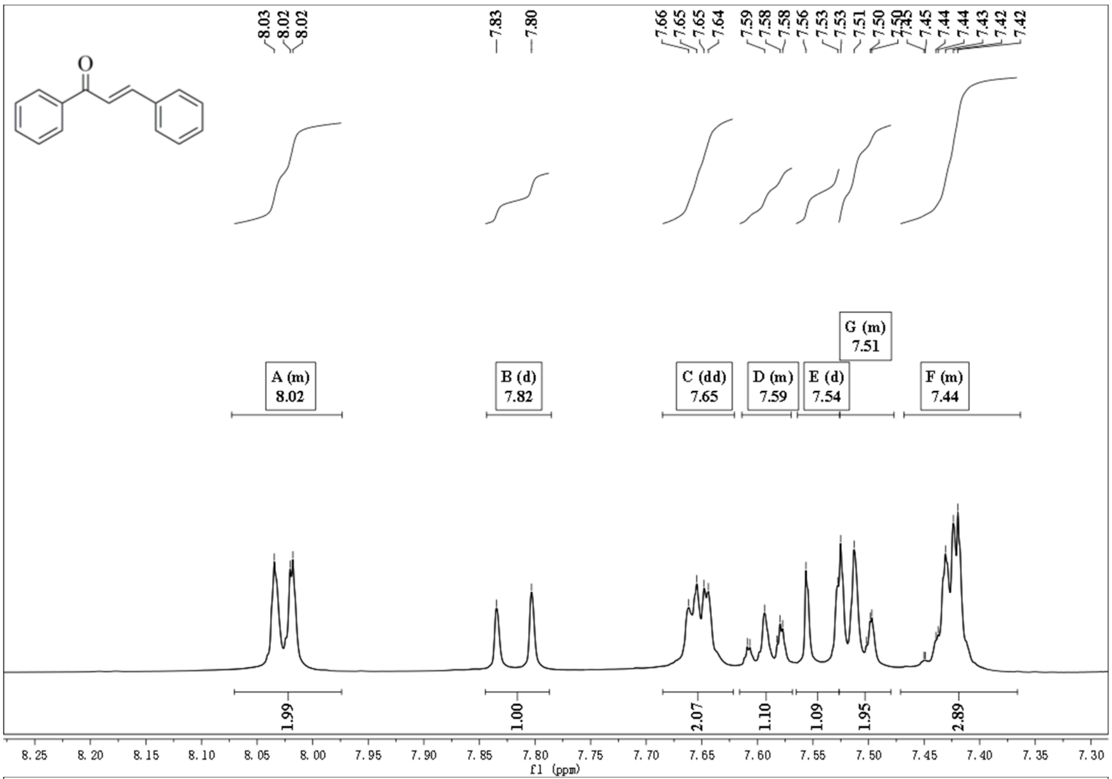
8.03
8.02
8.02
7.83
7.80
7.66
7.65
7.65
7.64
7.59
7.58
7.58
7.56
7.53
7.53
7.51
7.50
7.45
7.44
7.44
7.43
7.42
7.42
O
A (m) 8.02
B (d) 7.82
C (dd) 7.65
D (m) 7.59
E (d) 7.54
G (m) 7.51
F (m) 7.44
1.99
1.00
2.07
1.10
1.09
1.95
2.89
8.25 8.20 8.15 8.10 8.05 8.00 7.95 7.90 7.85 7.80 7.75 7.70 7.65 7.60 7.55 7.50 7.45 7.40 7.35 7.30
f1 (ppm)

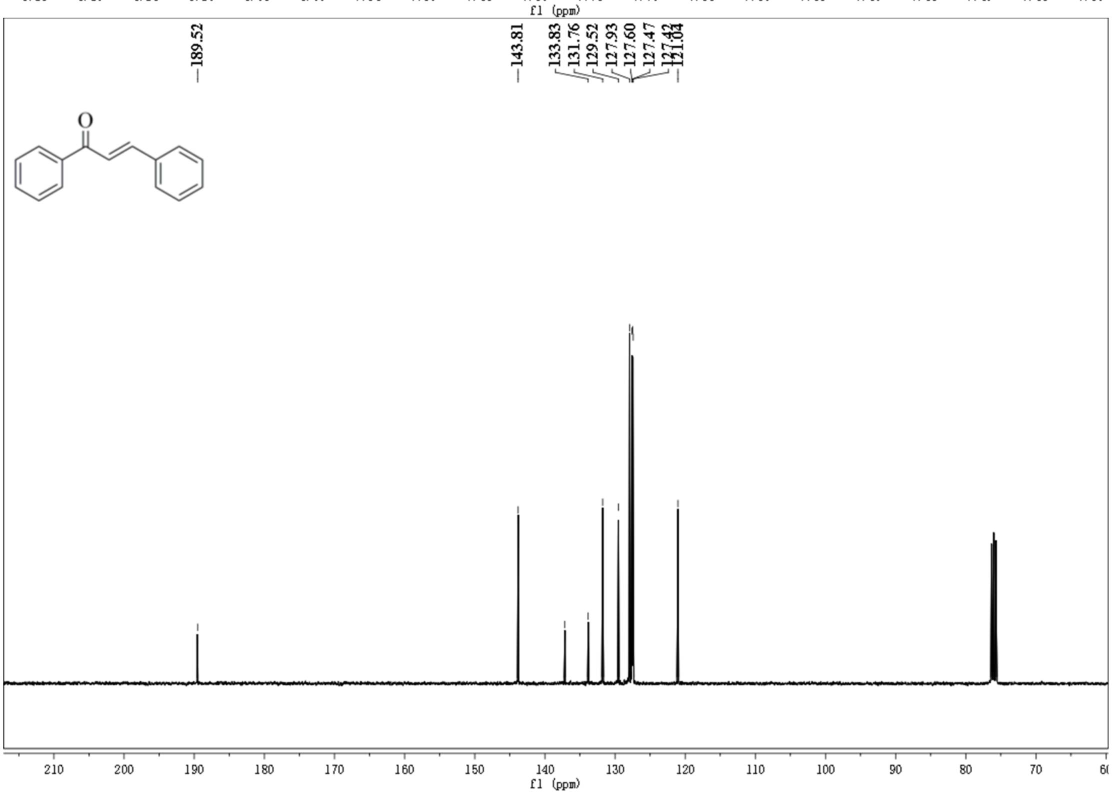
189.52
143.81
133.83
131.76
129.52
127.93
127.60
127.47
O
210 200 190 180 170 160 150 140 130 120 110 100 90 80 70
f1 (ppm)

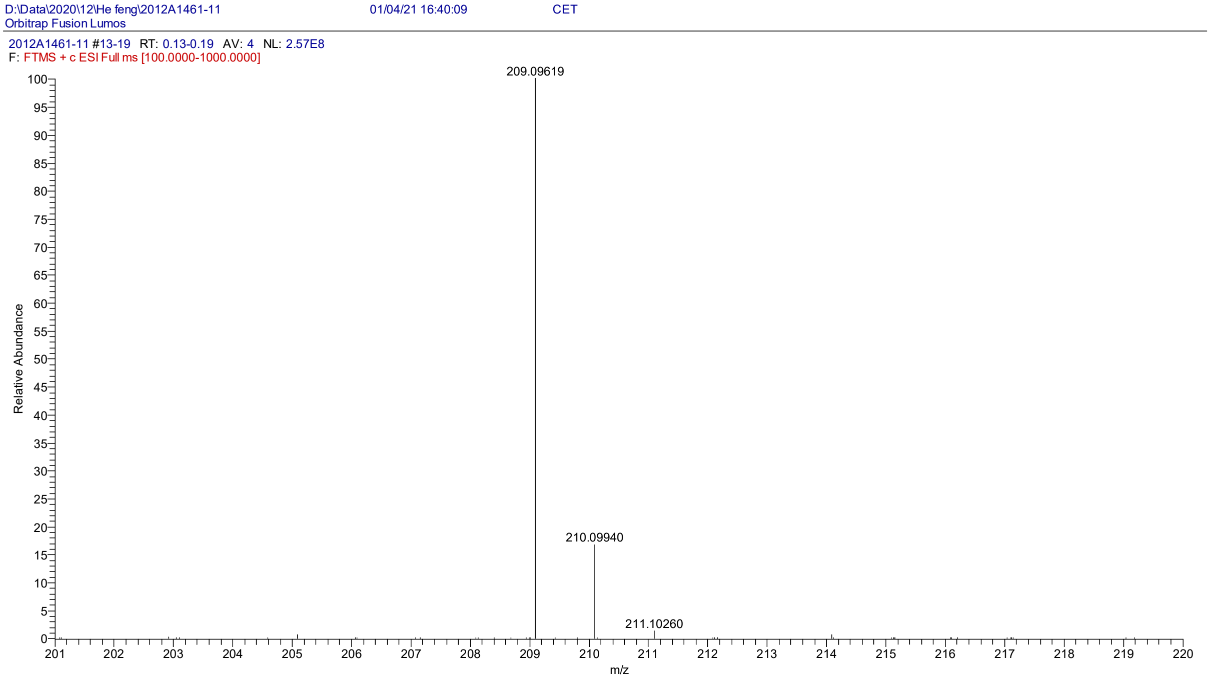

Figure S11. ^{1}H, ^{13}C NMR spectra and ESI-MS analysis for compound **7**.

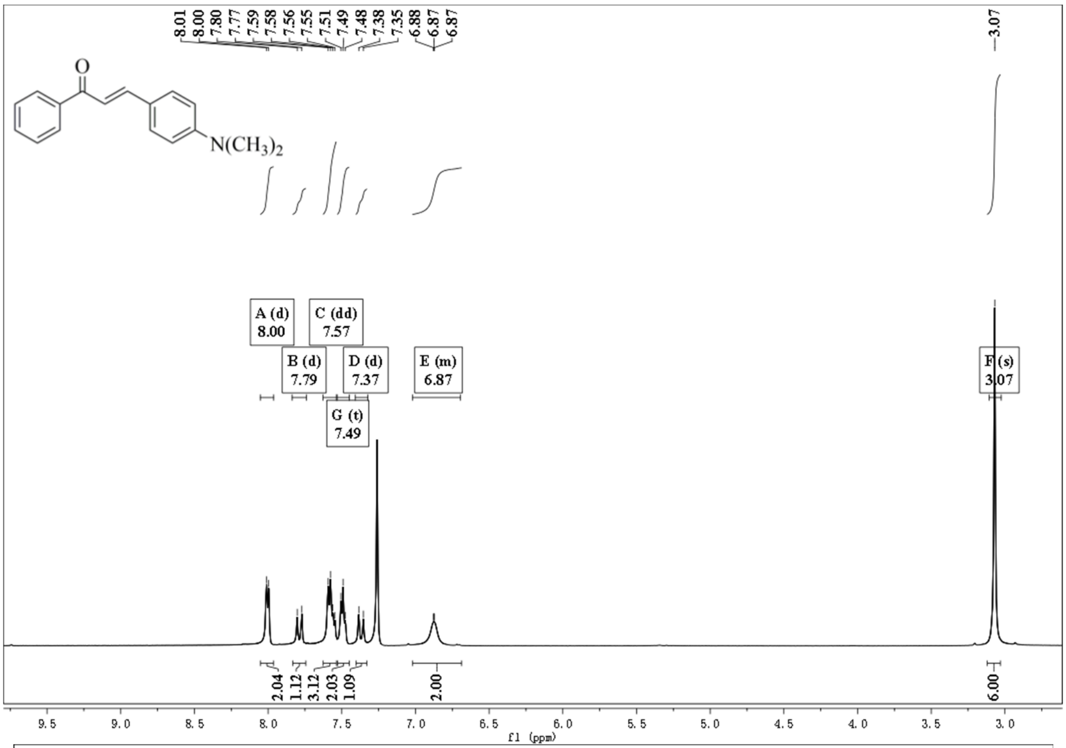
8.01
8.00
7.80
7.77
7.59
7.58
7.56
7.55
7.51
7.49
7.48
7.38
7.35
6.88
6.87
6.87
3.07
O
N(CH3)2
A (d)
8.00
C (dd)
7.57
B (d)
7.79
D (d)
7.37
E (m)
6.87
F (s)
3.07
G (t)
7.49
2.04
1.12
3.12
2.03
1.09
2.00
6.00
9.5
9.0
8.5
8.0
7.5
7.0
6.5
6.0
5.5
5.0
4.5
4.0
3.5
3.0
f1 (ppm)

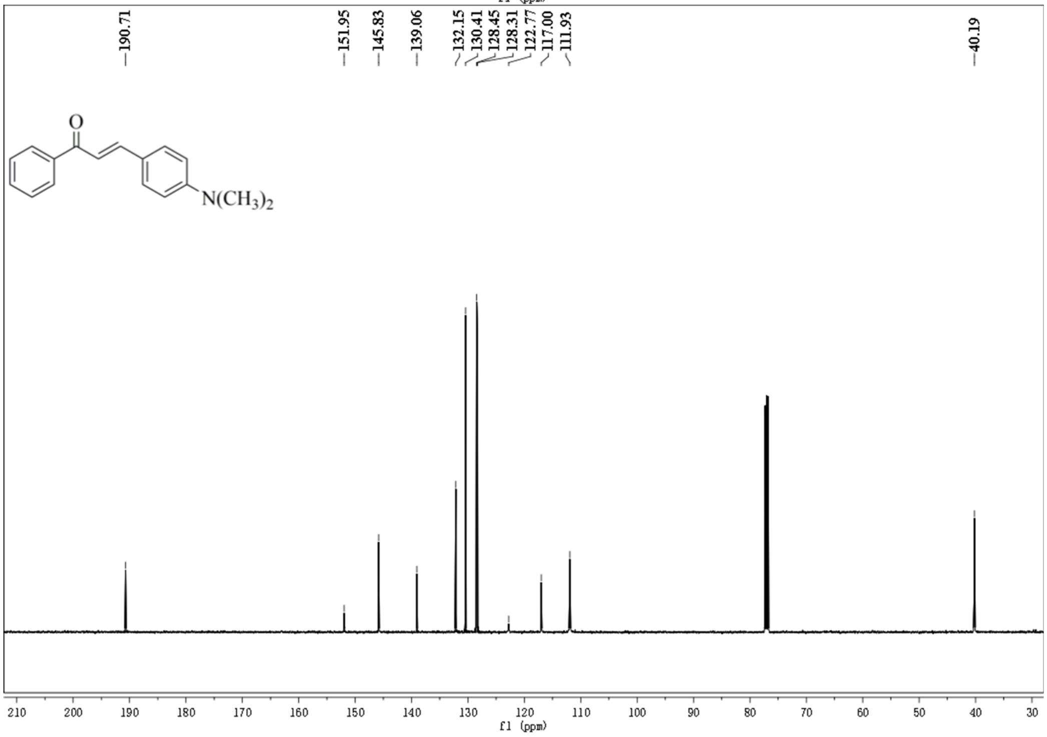
190.71
151.95
145.83
139.06
132.15
130.41
128.45
128.31
122.77
117.00
111.93
40.19
O
N(CH3)2
210
200
190
180
170
160
150
140
130
120
110
100
90
80
70
60
50
40
30
f1 (ppm)

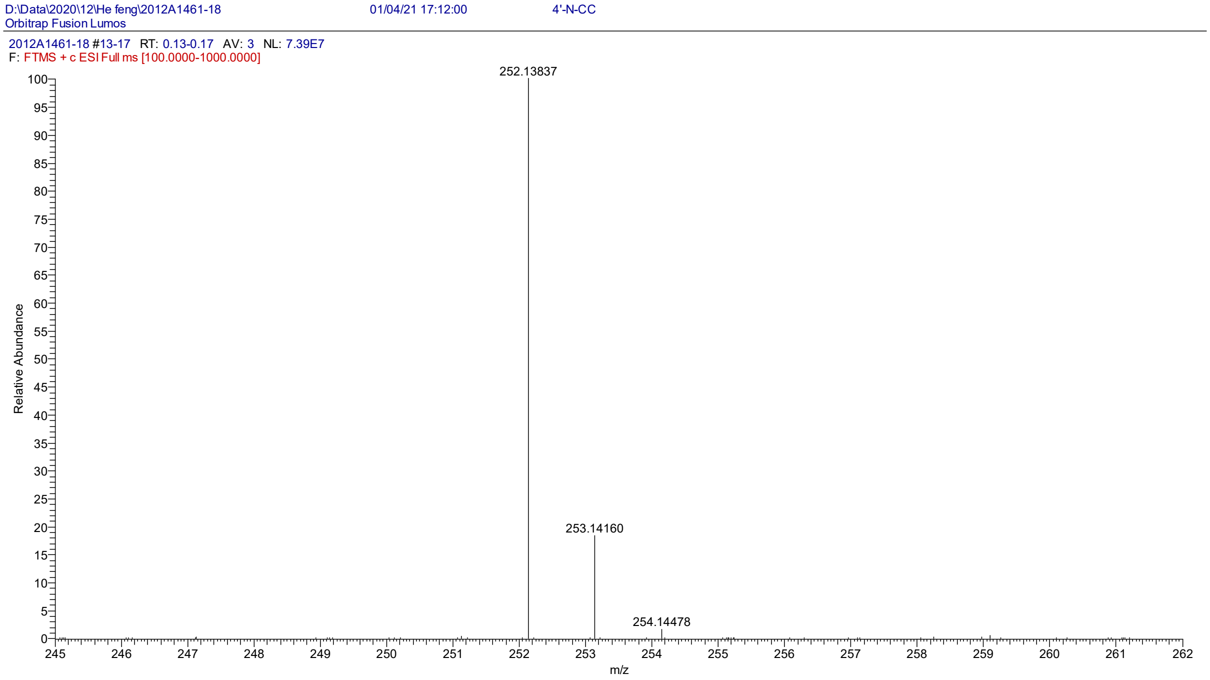

Figure S12. ^{1}H, ^{13}C NMR spectra and ESI-MS analysis for compound **8**.

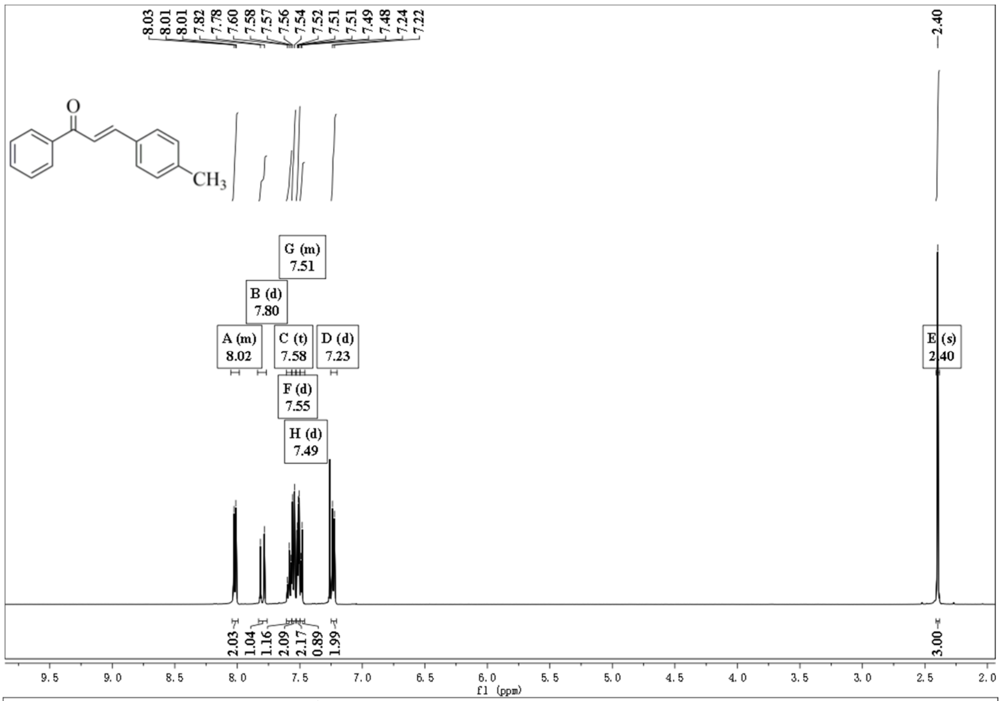
8.03
8.01
8.01
7.82
7.78
7.60
7.58
7.57
7.56
7.54
7.52
7.51
7.51
7.49
7.48
7.24
7.22
2.40
CH3
G (m)
7.51
B (d)
7.80
A (m)
8.02
C (t)
7.58
D (d)
7.23
F (d)
7.55
H (d)
7.49
E (s)
2.40
2.03
1.04
1.16
2.09
2.17
0.89
1.99
3.00
f1 (ppm)

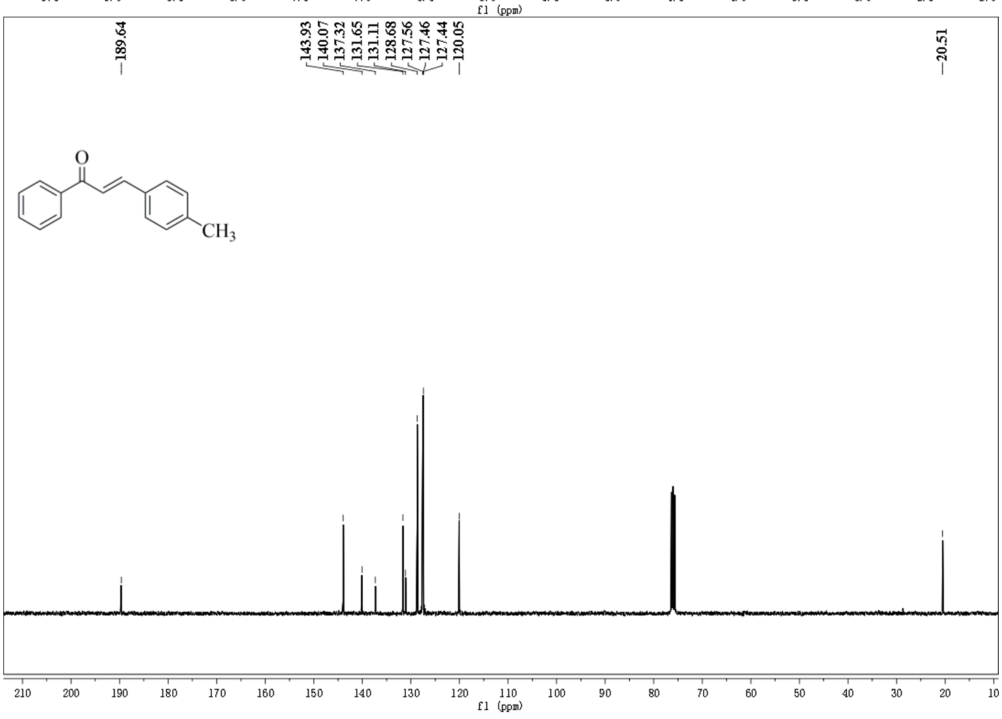
189.64
143.93
140.07
137.32
131.65
131.11
128.68
127.56
127.46
127.44
120.05
20.51
CH3
f1 (ppm)

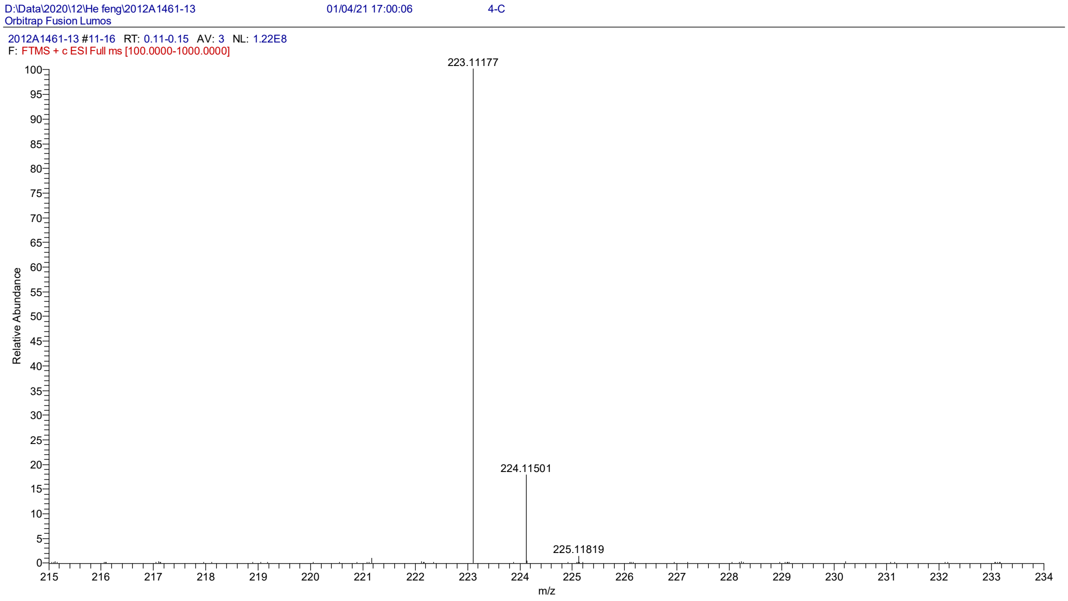

Figure S13. ^{1}H, ^{13}C NMR spectra and ESI-MS analysis for compound **9**.

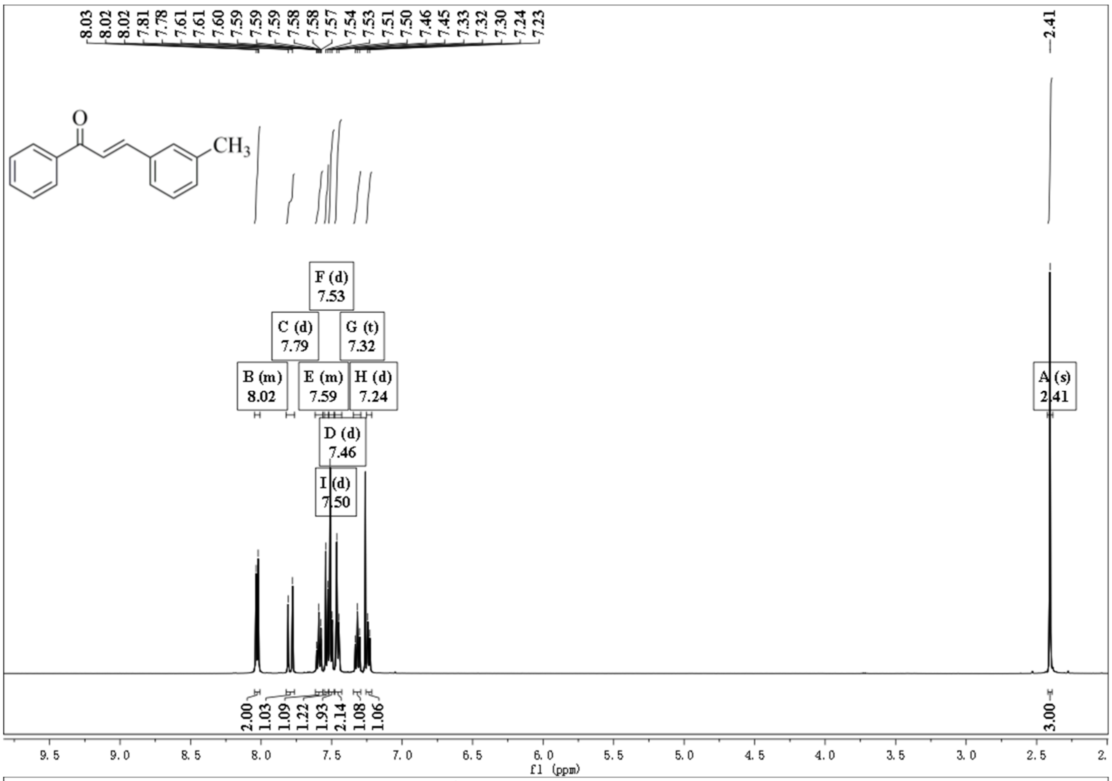
8.03 8.02 8.02 7.81 7.78 7.61 7.61 7.60 7.59 7.59 7.59 7.58 7.58 7.57 7.54 7.53 7.51 7.50 7.46 7.45 7.33 7.32 7.30 7.24 7.23
2.41
CH3
F (d) 7.53
C (d) 7.79
G (t) 7.32
B (m) 8.02
E (m) 7.59
H (d) 7.24
A (s) 2.41
D (d) 7.46
I (d) 7.50
2.00 1.03 1.09 1.22 1.93 2.14 1.08 1.06
3.00
f1 (ppm)

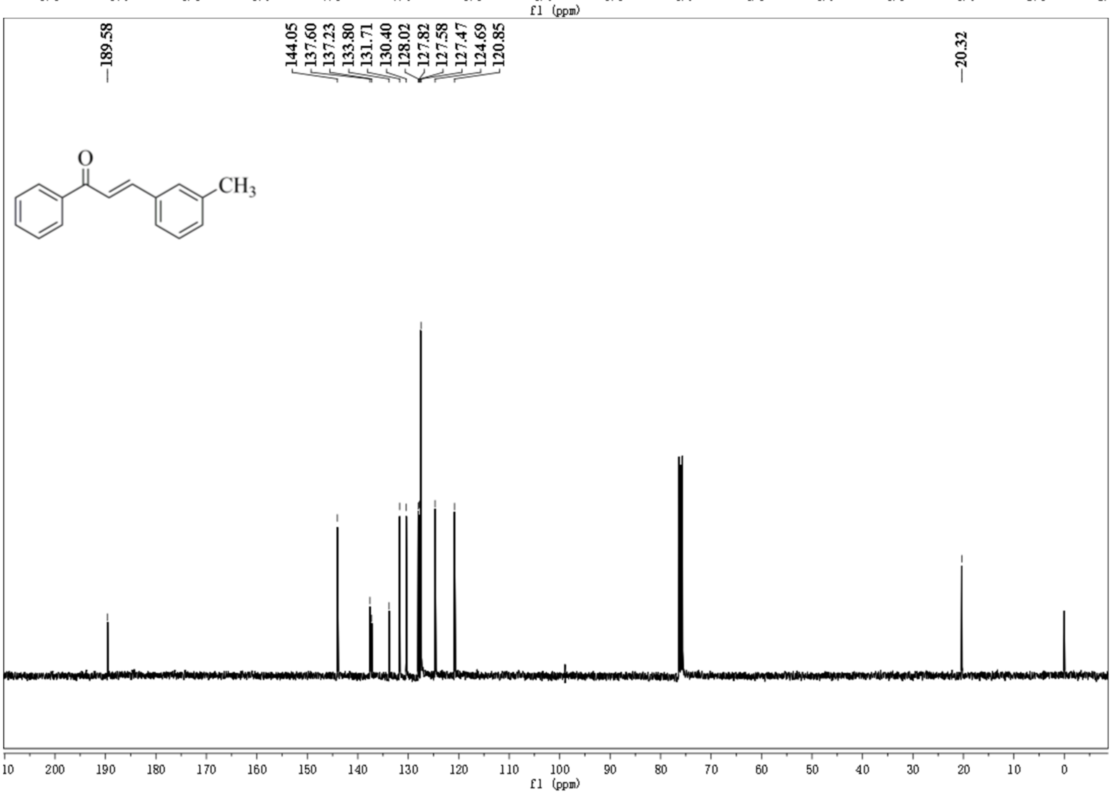
189.58
144.05 137.60 137.23 133.80 131.71 130.40 128.02 127.82 127.58 127.47 124.69 120.85
20.32
CH3
f1 (ppm)

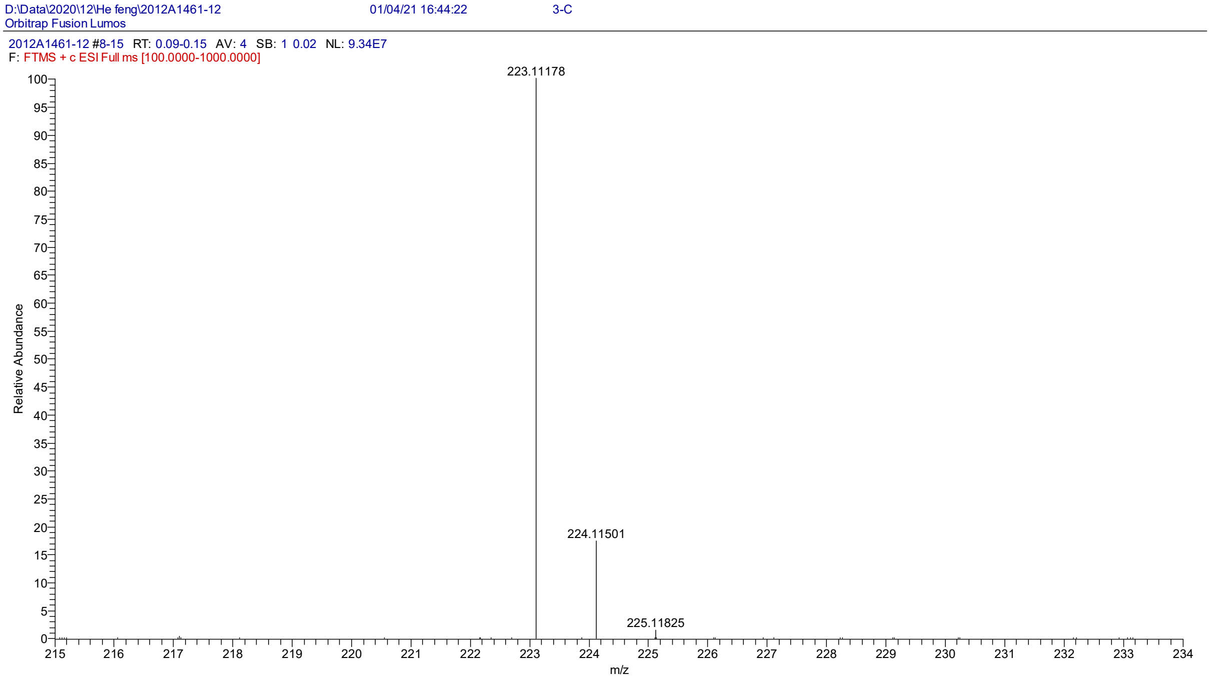

Figure S14. ^{1}H, ^{13}C NMR spectra and ESI-MS analysis for compound **10**.

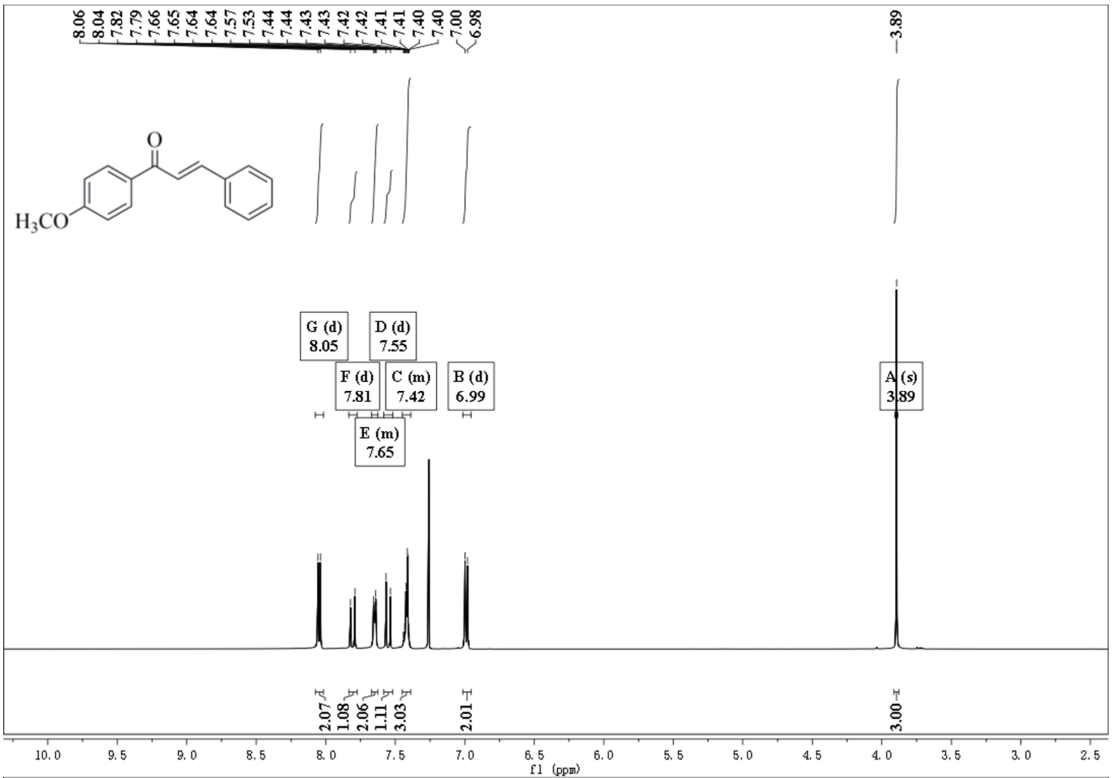
8.06
8.04
7.82
7.79
7.66
7.65
7.64
7.64
7.57
7.53
7.44
7.44
7.43
7.43
7.42
7.42
7.41
7.41
7.40
7.40
7.00
6.98
3.89
O
H3CO
G (d) 8.05
D (d) 7.55
F (d) 7.81
C (m) 7.42
B (d) 6.99
A (s) 3.89
E (m) 7.65
2.07
1.08
2.06
1.11
3.03
2.01
3.00
f1 (ppm)

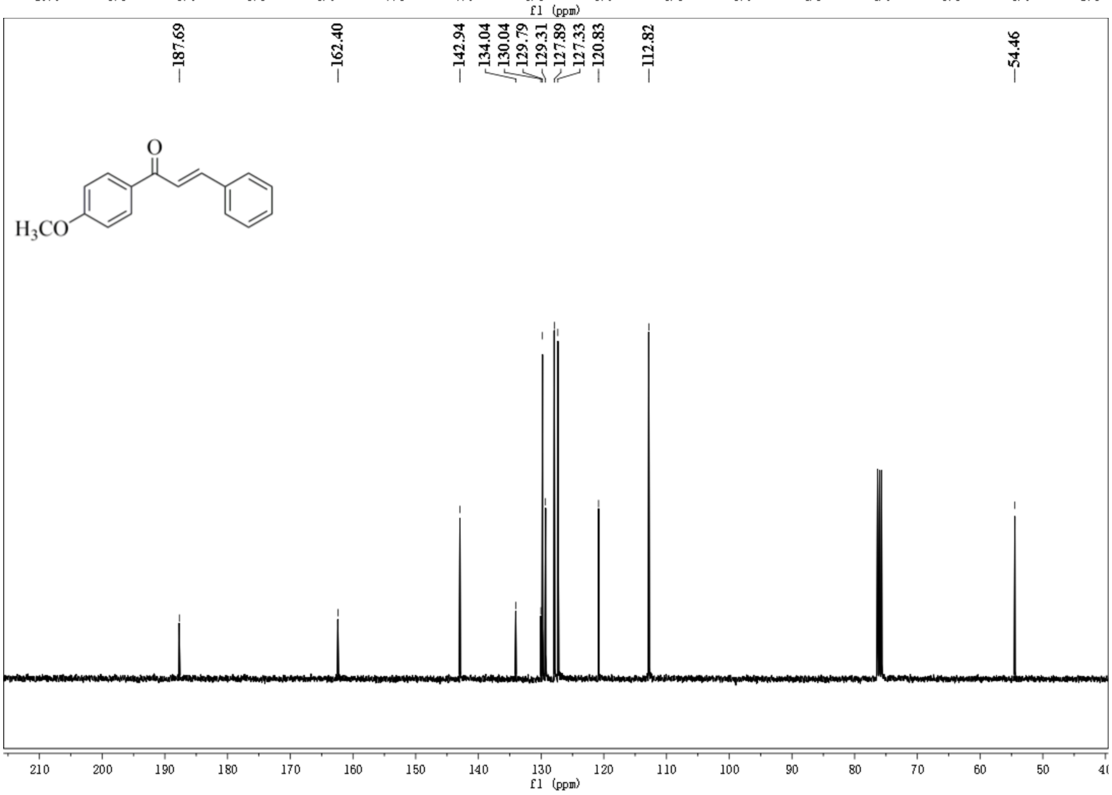
187.69
162.40
142.94
134.04
130.04
129.79
129.31
127.89
127.33
120.83
112.82
54.46
O
H3CO
f1 (ppm)

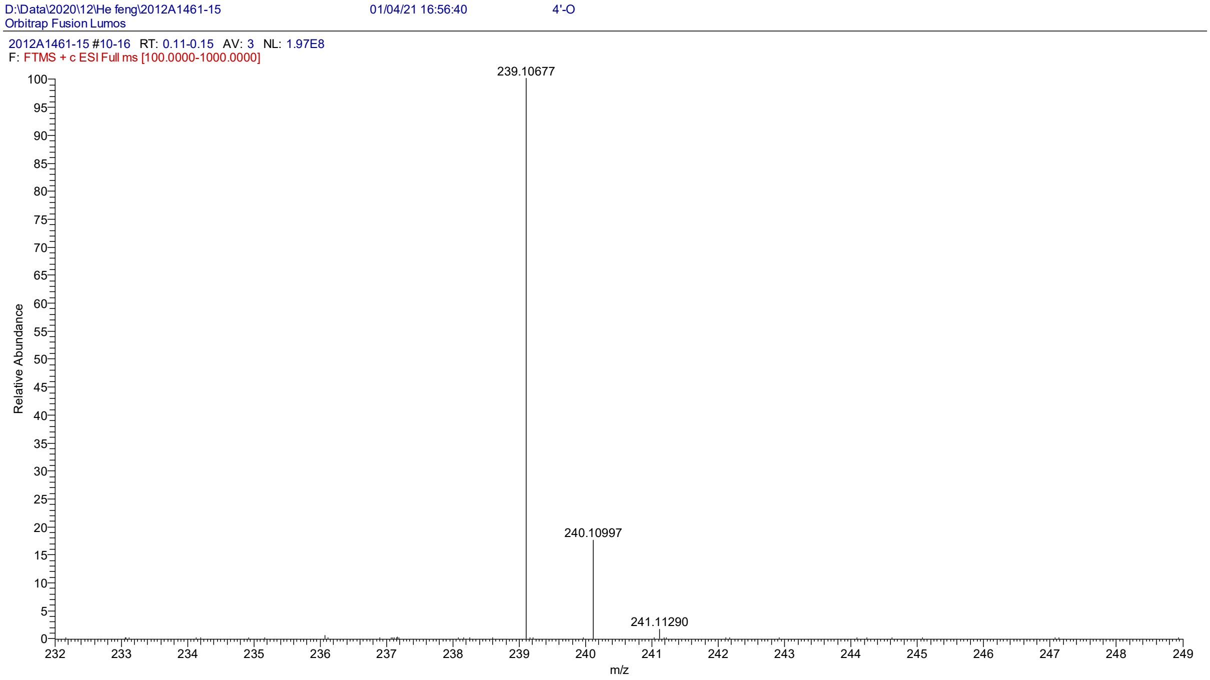

Figure S15. ^{1}H, ^{13}C NMR spectra and ESI-MS analysis for compound **11**.
